# Supplementary material for: Relation of connectome topology to brain volume across 103 mammalian species
Source: PLoS Biol. 2024 Feb 5;22(2):e3002489. doi: 10.1371/journal.pbio.3002489 (PMC10868790; doi:10.1371/journal.pbio.3002489)
Supplement: S1 File — (PDF) [file pbio.3002489.s001.pdf]

S1. Replication on differently thresholded networks

Table A in S1 File. The table contains correlation coefficients and p-values of the analysis described in the main text at the result section "*Scaling laws of modular organization*" replicated on networks built with different thresholds, so that they have different densities (see also Methods, "*Mammalian connectome construction*"). The analysis has been replicated also using gray and white matter volumes as dependent variable, instead of the whole brain volume, and correlation coefficients and p-values have been reported in the third and fourth column of the table.

	MODULARITY MEASURES	Brain Volume (log10)		Grey Matter (log10)		White Matter (log10)	
		p_{val}	ρ	p_{val}	ρ	p_{val}	ρ
thr = 0	Intra-module density	<0.0001	0.47	<0.0001	0.47	<0.0001	0.46
	ρ(CC,weight)	<0.0001	0.60	<0.0001	0.59	<0.0001	0.61
	ρ(CC,cost)	<0.0001	0.62	<0.0001	0.61	<0.0001	0.63
	ρ(CC,ED)	<0.0001	-0.44	<0.0001	-0.43	<0.0001	-0.46
	Long-dist betw. modules	<0.0001	0.35	<0.0001	0.35	<0.0001	0.33
	Inter-hemispheric modules	0.29	0.08	0.32	0.07	0.17	0.10
thr = 0.05	Intra-module density	<0.0001	0.41	<0.0001	0.41	<0.0001	0.40
	ρ(CC,weight)	<0.0001	0.55	<0.0001	0.54	<0.0001	0.55
	ρ(CC,cost)	<0.0001	0.55	<0.0001	0.54	<0.0001	0.55
	ρ(CC,ED)	<0.0001	-0.44	<0.0001	-0.43	<0.0001	-0.46
	Long-dist betw. modules	0.0017	0.22	0.0017	0.22	0.0028	0.21
	Inter-hemispheric modules	0.81	0.02	0.88	0.01	0.48	0.05
thr = 0.1	Intra-module density	<0.0001	0.45	<0.0001	0.44	<0.0001	0.44
	ρ(CC,weight)	<0.0001	0.60	<0.0001	0.60	<0.0001	0.60
	ρ(CC,cost)	<0.0001	0.61	<0.0001	0.60	<0.0001	0.60
	ρ(CC,ED)	<0.0001	-0.47	<0.0001	-0.46	<0.0001	-0.50
	Long-dist betw. modules	<0.0001	0.28	<0.0001	0.28	<0.0001	0.28
	Inter-hemispheric modules	0.82	0.02	0.85	0.01	0.44	0.04
thr = 0.15	Intra-module density	<0.0001	0.43	<0.0001	0.43	<0.0001	0.41
	ρ(CC,weight)	<0.0001	0.61	<0.0001	0.60	<0.0001	0.61
	ρ(CC,cost)	<0.0001	0.62	<0.0001	0.61	<0.0001	0.62
	ρ(CC,ED)	<0.0001	-0.42	<0.0001	-0.41	<0.0001	-0.45
	Long-dist betw. modules	<0.0001	0.30	<0.0001	0.30	<0.0001	0.28
	Inter-hemispheric modules	0.50	0.05	0.53	0.05	0.29	0.08

Table B in S1 File. The table contains correlation coefficients and p-values of the analysis described in the main text at the result section "*Scaling laws of communication efficiency*" replicated on networks built with different thresholds, so that they have different densities (see also Methods, "*Mammalian connectome construction*"). The analysis has been replicated also using gray and white matter volumes as dependent variable, instead of the whole brain volume, and correlation coefficients and p-values have been reported in the third and fourth column of the table.

COMMUNICATION MEASURES		Brain Volume (log10)		Grey Matter (log10)		White Matter (log10)	
		p_{val}	ρ	p_{val}	ρ	p_{val}	ρ
thr =0	ρ(ED, SPE)	<0.0001	-0.53	<0.0001	-0.52	<0.0001	-0.51
	ρ(ED, NSI)	<0.0001	-0.54	<0.0001	-0.53	<0.0001	-0.54
	ρ(ED, CMY)	<0.0001	-0.62	<0.0001	-0.61	<0.0001	-0.63
thr =0.05	ρ(ED, SPE)	<0.0001	-0.55	<0.0001	-0.54	<0.0001	-0.53
	ρ(ED, NSI)	<0.0001	-0.53	<0.0001	-0.53	<0.0001	-0.54
	ρ(ED, CMY)	<0.0001	-0.55	<0.0001	-0.55	<0.0001	-0.57
thr =0.1	ρ(ED, SPE)	<0.0001	-0.56	<0.0001	-0.56	<0.0001	-0.54
	ρ(ED, NSI)	<0.0001	-0.55	<0.0001	-0.55	<0.0001	-0.56
	ρ(ED, CMY)	<0.0001	-0.61	<0.0001	-0.60	<0.0001	-0.62
thr =0.15	ρ(ED, SPE)	<0.0001	-0.56	<0.0001	-0.56	<0.0001	-0.54
	ρ(ED, NSI)	<0.0001	-0.54	<0.0001	-0.53	<0.0001	-0.55
	ρ(ED, CMY)	<0.0001	-0.60	<0.0001	-0.59	<0.0001	-0.61
