# Supplementary material for: Relation of connectome topology to brain volume across 103 mammalian species
Source: PLoS Biol. 2024 Feb 5;22(2):e3002489. doi: 10.1371/journal.pbio.3002489 (PMC10868790; doi:10.1371/journal.pbio.3002489)
Supplement: S2 File — (PDF) [file pbio.3002489.s002.pdf]

S2. Modularity analysis on single-scale partitions

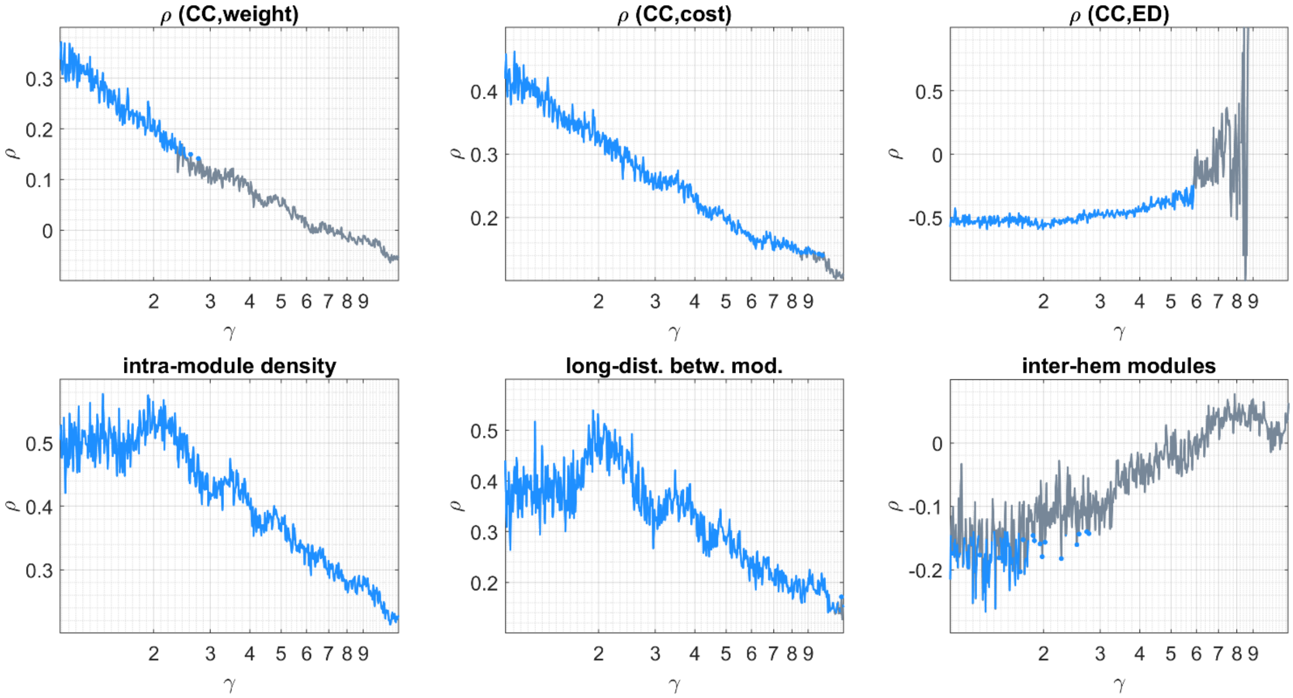

Figure A in S2 File. Each panel of the figure shows for each γ (x-axis) the correlation coefficients ρ (y-axis) of the Spearman correlation computed between the modularity indices (title of the panels) and brain volume. Statistically significant ρ have been highlighted in blue.
