# Supplementary material for: Relation of connectome topology to brain volume across 103 mammalian species
Source: PLoS Biol. 2024 Feb 5;22(2):e3002489. doi: 10.1371/journal.pbio.3002489 (PMC10868790; doi:10.1371/journal.pbio.3002489)
Supplement: S3 File — (PDF) [file pbio.3002489.s003.pdf]

S3. Validation of the results on null models

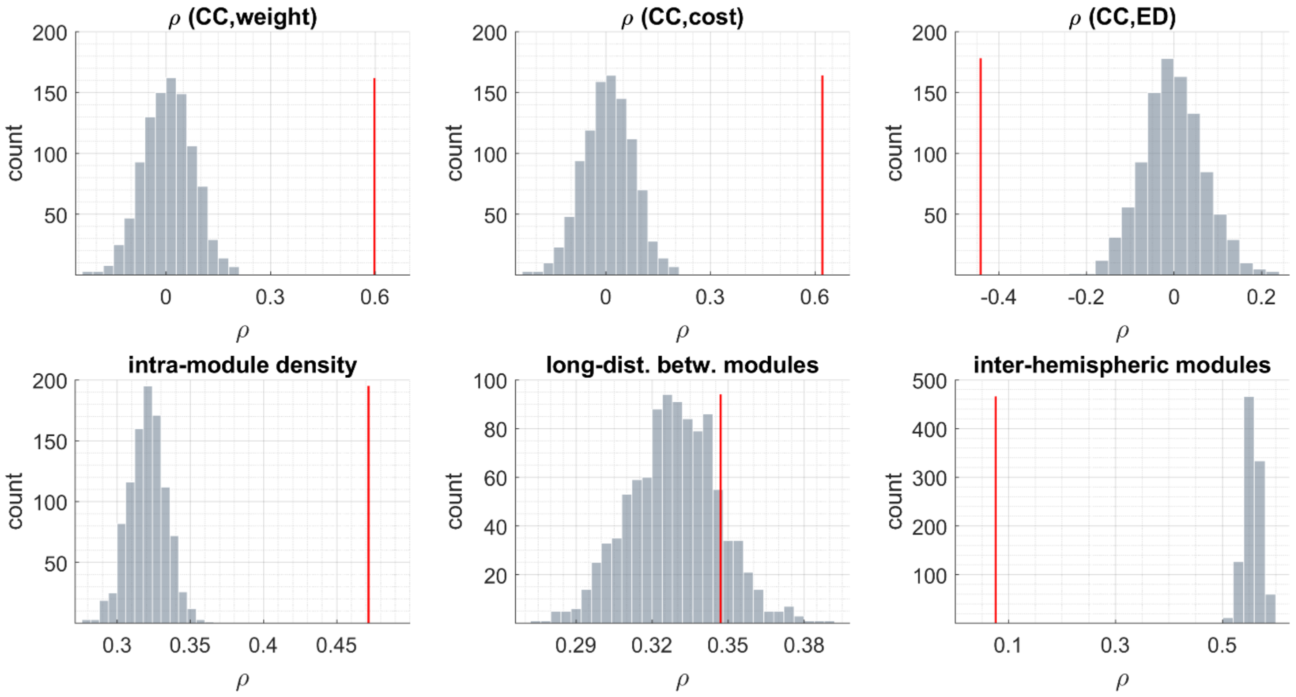

Figure A in S3 File. The same indices reported in Figure 3 of the main text, were computed on co-classification matrices and consensus partitions where entries have been randomly permuted (the permutation was consistent across mammals) 1000 times. This null model randomly permuted co-classification probability between pairs of nodes, while preserving the overall co-classification distribution. As for the consensus partitions, it randomly permuted module allegiance, while preserving the number and size of modules. Each panel of the figure reports the distribution of the correlation coefficients obtained under the null model (grey) and the actual correlation coefficient derived from the observed data (red line). All the observed indices were statistically different from those obtained in the null case.

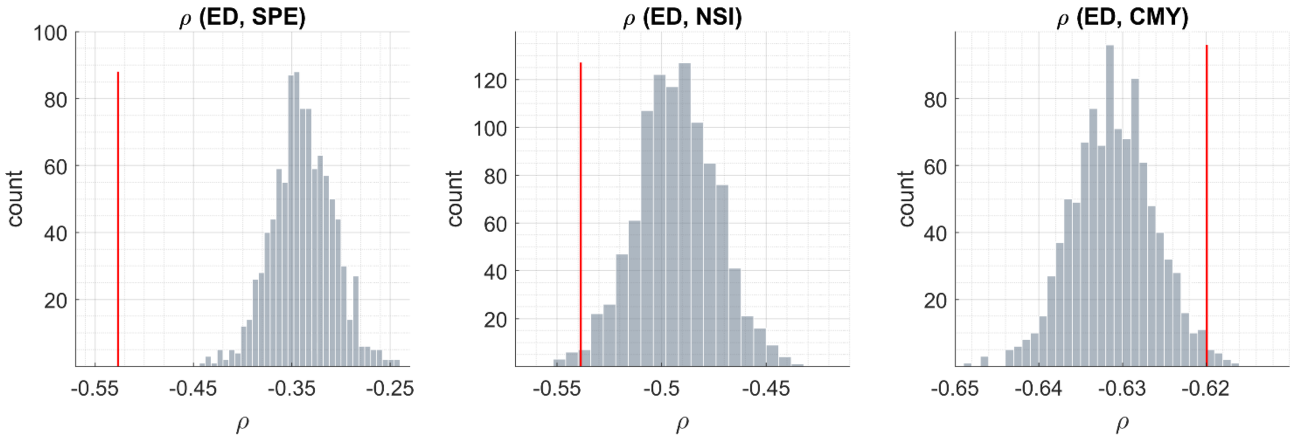

Figure B in S3 File. Communication measures and their correlation with the Euclidean distance between nodes, were computed for each mammal on 1000 surrogate random networks obtained by preserving the original geometry and degree distribution of the 201 brain networks. Surrogate networks have been generated by binning edges by distance (each bin contained an equal number of edges), and then shuffling edges within bins. Results are reported in this figure, where in each panel we show the distribution of the

correlation coefficients obtained under the null model (grey) and those observed in the actual model (red line), for the three communication measures. All the correlation coefficients obtained for empirical data were statistically different from those obtained in the null case.
