# Supplementary material for: Relation of connectome topology to brain volume across 103 mammalian species
Source: PLoS Biol. 2024 Feb 5;22(2):e3002489. doi: 10.1371/journal.pbio.3002489 (PMC10868790; doi:10.1371/journal.pbio.3002489)
Supplement: S4 File — (PDF) [file pbio.3002489.s004.pdf]

S4. Validation of the results on sub samples of the dataset comprising only one animal per species

Table A in S4 File. The table contains correlation coefficients of the analysis presented in the main text under the section "*Scaling laws of modular organization*" replicated on a sub sample of the dataset containing only one animal per species. One animal per species was randomly selected a thousand times and the correlations between modularity indices and brain volume were computed each time, so that in the table we report mean and standard deviation of the correlation coefficients. Indices for which the correlations were not statistically significant have been indicated with a red asterisk.

	MODULARITY MEASURES	Brain Volume (log10)	Grey Matter (log10)	White Matter (log10)
		ρ	ρ	ρ
thr = 0	Intra-module density	0.51 ± 0.03	0.51 ± 0.03	0.51 ± 0.03
	ρ(CC,weight)	0.68 ± 0.02	0.68 ± 0.02	0.7 ± 0.02
	ρ(CC,cost)	0.71 ± 0.02	0.70 ± 0.02	0.72 ± 0.02
	ρ(CC,ED)	-0.49 ± 0.03	-0.49 ± 0.03	-0.5 ± 0.03
	Long-dist betw. modules	0.37 ± 0.04	0.36 ± 0.04	0.36 ± 0.04
	Inter-hemispheric modules*	0.09 ± 0.03	0.09 ± 0.03	0.11 ± 0.03
