# Supplementary material for: Relation of connectome topology to brain volume across 103 mammalian species
Source: PLoS Biol. 2024 Feb 5;22(2):e3002489. doi: 10.1371/journal.pbio.3002489 (PMC10868790; doi:10.1371/journal.pbio.3002489)
Supplement: S5 File — (PDF) [file pbio.3002489.s005.pdf]

S5. Inter- and intra-hemispheric connectivity across brain volume

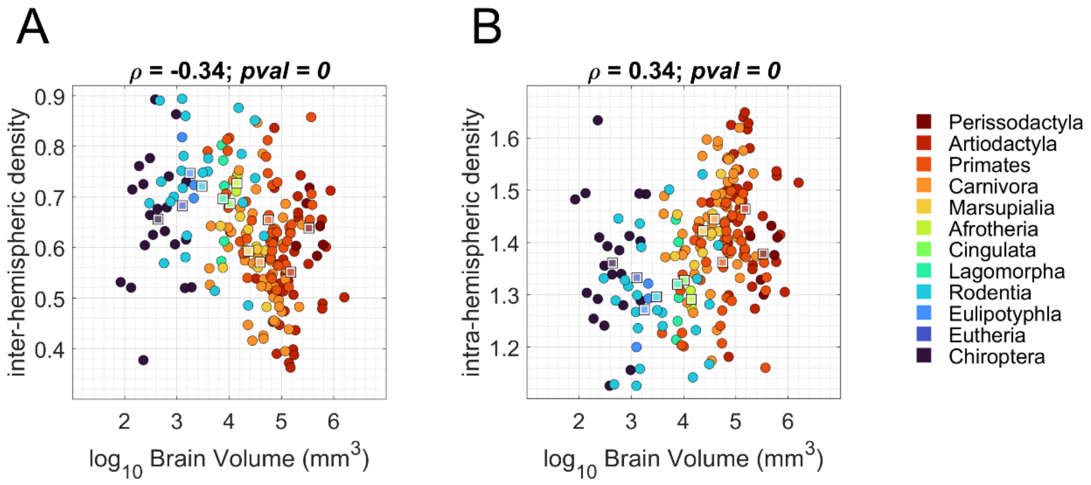

Figure A in S5 File. We represented the distributions of brain volume (x-axis) versus inter-hemispheric density (A) and intra-hemispheric density (B). Both densities have been normalized in each mammal by the overall network density. In each panel, the different taxonomies are highlighted through different colors and the median values of their respective measures are reported with squares of the same color.
