# Supplementary material for: Relation of connectome topology to brain volume across 103 mammalian species
Source: PLoS Biol. 2024 Feb 5;22(2):e3002489. doi: 10.1371/journal.pbio.3002489 (PMC10868790; doi:10.1371/journal.pbio.3002489)
Supplement: S6 File — (PDF) [file pbio.3002489.s006.pdf]

S6. Controlling correlations with similarity between species and orders

As shown in Figure 2D, brin volume and orders are collinear: on average different orders display different brain volumes, although the inter-order variability remains high above all among Chiroptera, Rodentia, Carnivora, Primates, and Artiodactyla, and brain volume overlaps across these orders. To confirm the robustness of our results and prove that connectome topology is shaped by brain volume, we carried out this further analysis where we recomputed the partial correlations between modularity/communication indices and brain volume controlling first for the similarity across species and then for orders. The results are shown in Figure S5 and S6, respectively.

The similarity across species, or phylogenetic distances, have been estimated stochastically for the MaMI database in [1] using a set of 10,000 phylogenetic trees inferred using a Bayesian approach [2]. For each tree, we computed the pairwise distance between species by adding the patristic distance along the tree branches. Then, we pruned distance matrices to match the species contained in the MaMI database. For more details see [1]. As a result we have 10,000 matrices containing phylogenetic distances among the species. By taking the centroid of these matrices and computing a Principal Component Analysis (PCA), we obtained coefficients that encode modes of covariance between species. We used the first three coefficients (explaining most of the variance in the PCA, see Figure A, panel A) as predictors in the partial correlation between modularity/communication indices and brain volume. As a result, we observed that the correlations documented in the main text hold. In fact, p-values and correlation coefficients (Figure A, panel B) remain consistent with those reports in Figures 3 and 4.

We repeated an analogous partial correlation analysis but this time controlling for taxonomy orders. Results are reported in the tables in Figure S6. All the correlations remain statistically significant (p-value<0.05), although the correlation coefficients are weaker. The only exception is given by the partial correlation between intra-module density and brain volume, that is non-significant.

Altogether, these results show that, while in some cases they are attenuated, the correlations documented in the main analysis hold. This demonstrates that phylogenetic distances and orders only partially explain the trends. Again, the fact that these trends are partially explained by phylogeny is not surprising given the collinearity observed in Figure 2D between brain volume and taxonomy.

REFERENCES

1. Faskowitz J., Puxeddu M.G., van den Heuvel M.P., Mišić B., Yovel Y., Assaf Y., Betzel R.F., and Sporns O. (2023). Connectome topology of mammalian brains and its relationship to taxonomy and phylogeny. Frontiers in Neuroscience. https://doi.org/10.3389/fnins.2022.1044372.
2. Upham, N. S., Esselstyn, J. A., and Jetz, W. (2019). Inferring the mammal tree: Species-level sets of phylogenies for questions in ecology, evolution, and conservation. PLoS Biology. 17:e3000494. doi: 10.1371/journal.pbio.3000494.

A

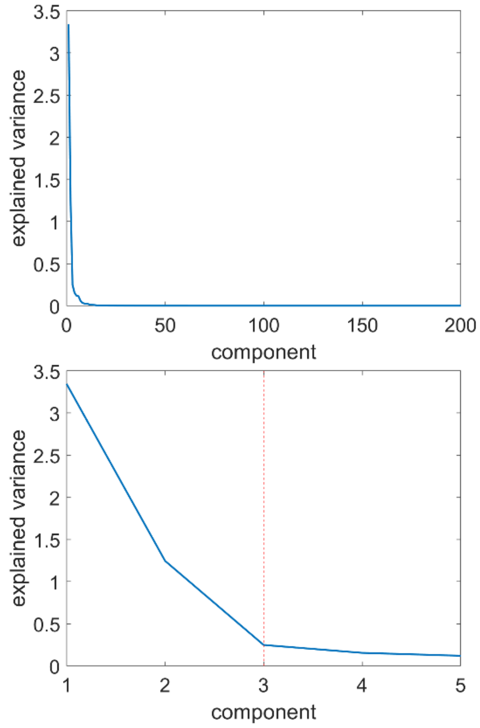

B

MODULARITY MEASURES		Brain Volume (log10)	
		p_{val}	ρ
thr = 0	Intra-module density	<0.0001	0.51
	ρ(CC,weight)	<0.0001	0.58
	ρ(CC,cost)	<0.0001	0.60
	ρ(CC,ED)	<0.0001	-0.44
	Long-dist betw. modules	<0.0001	0.37
	Inter-hemispheric modules	0.35	0.07

COMMUNICATION MEASURES		Brain Volume (log10)	
		p_{val}	ρ
thr =0	ρ(ED, SPE)	<0.0001	-0.44
	ρ(ED, NSI)	<0.0001	-0.44
	ρ(ED, CMY)	<0.0001	-0.56

Figure A in S6 File. Results of the partial correlations between modularity/communication indices and brain volume using as predictors the similarity among species. In panel A we report the explained variance of the PCA, whereas in panel B the correlation coefficients and p-values. The underlying data for this figure can be found in S1_data.xlsx.

MODULARITY MEASURES		Brain Volume (log10)	
		p_{val}	ρ
thr = 0	Intra-module density	0.64	0.03
	ρ(CC,weight)	0.03	0.15
	ρ(CC,cost)	0.0094	0.19
	ρ(CC,ED)	0.0112	-0.18
	Long-dist betw. modules	0.0128	0.18
	Inter-hemispheric modules	0.2398	0.08

COMMUNICATION MEASURES		Brain Volume (log10)	
		p_{val}	ρ
thr =0	ρ(ED, SPE)	0.0002	-0.27
	ρ(ED, NSI)	0.011	-0.19
	ρ(ED, CMY)	<0.0001	-0.32

Figure B in S6 File. Results of the partial correlations between modularity/communication indices and brain volume using as predictors the taxonomy orders.
