# Supplementary material for: Relation of connectome topology to brain volume across 103 mammalian species
Source: PLoS Biol. 2024 Feb 5;22(2):e3002489. doi: 10.1371/journal.pbio.3002489 (PMC10868790; doi:10.1371/journal.pbio.3002489)
Supplement: Attachment — Submitted filename: response_to_reviewers.docx [file pbio.3002489.s008.docx]

REVIEWER 1

This is a comparative diffusion MRI-based connectomics study that evaluates the relationship between network modularity and brain volumes. The authors analyzed diffusion MRI data acquired from a large number (102) of mammalian species. They first replicated the known scaling law regarding the relationship between gray matter and white matter volume. They then demonstrated how inter-species differences in brain volume are related to the network properties of the connectome.

I have identified both positive and negative points about the manuscript. Below, I will highlight my comments.

The comprehensive analysis of diffusion MRI data from many mammalian species is an impressive aspect of this study. Additionally, it is nice to see the replication of the scaling law in a neuroimaging dataset acquired from various species. Although this scaling law is widely known, its demonstration in neuroimaging is an important contribution, as it establishes proof that neuroimaging is a useful method for understanding evolutionary principles.

The visualizations presented in this paper are clear, and the analyses are conducted by an experienced group in network neuroscience. The authors have made their MATLAB codes available to the public, which is a good practice for ensuring the reproducibility of findings.

While I don't have significant concerns regarding the scientific goals and reproducibility of this work, I do have some concerns about the writing style and interpretation of findings.

We thank the reviewer for the overall positive feedback on our work. We will respond to specific concerns in the following paragraphs.

------------------------------------------------------------------------------------------------------------------------

The writing style assumes that readers already possess knowledge about network analysis in connectomics. While this may be suitable for specialized journals in network neuroscience, I believe this style is not appropriate for the current journal, which targets a general readership in biology. The authors introduce many concepts in network analysis, such as modularity and communication efficiency. However, since the introduction does not sufficiently explain the importance of these concepts to general readers, the main results may be difficult to understand for readers who are not experts in network neuroscience. Therefore, if the authors aim to publish this work in this journal, they need to thoroughly revise the paper to make it more accessible to general readers without a strong background in network analysis.

We thank the reviewer for this suggestion. To improve the readability of the paper we added a glossary box where we fix the meaning of fundamental terms and concepts used in network neuroscience, like modularity and network communication efficiency. This will help readers who are not familiar with network neuroscience to navigate the manuscript.

Results, page 2:

*“Table 1. Glossary. Fundamental concepts and terms used in network neuroscience. More technical details about the network topological measures investigated are provided in the Methods section.”*

| *Networks and brain networks* | *A network is a mathematical object used to model complex systems, often biological, like the brain. Under the network conceptualization, the fundamental units of a system (cortical areas in the case of the brain) and their mutual interactions are formalized as a set of nodes connected (or not) by edges.* |
| --- | --- |
| *Network topology* | *Relation between nodes and edges. Network topology can be investigated through measures developed in the field of graph theory, like modularity and communication efficiency.* |
| *Network modules* | *Empirical networks, such as brain networks, often exhibit a non-random distribution of edges that results in distinct groups of highly interconnected nodes, also named modules, communities, or clusters. Network modules can be defined mathematically by optimizing modularity, a function that measures the strength of the division of the network into modules by comparing the within-module connectivity with a null model.* |
| *Network Communication* | *How nodes are integrated through sequences of edges, e.g., paths. Models of network communication range from routing protocols to diffusive processes. Cardinal measures falling in this range are: (i) shortest path efficiency, which captures the length of optimally efficient routes; (ii) search information, or the amount of information required from a random walker to travel along the shortest path, which estimates the accessibility of shortest routes in the network; (iii) communicability, or the sum of the lengths of all the possible paths connecting two nodes, which quantifies diffusive broadcasting.* |

------------------------------------------------------------------------------------------------------------------------

I am concerned that the authors did not acknowledge the works of Karl Zilles and others, who demonstrated a strong relationship between brain volume and gyrification. Without considering gyrification as a confounding factor, interpreting the present results becomes challenging. It is widely reported that tractography can be biased by cortical folding patterns. Hence, I suspect that some inter-species differences in network properties may simply reflect biases in tractography due to sulci/gyri, rather than true biological differences. Conducting control analyses that account for gyrification as a confounding factor is essential to provide confidence in interpreting the results.

We thank the reviewer for pointing out that gyrification may be intrinsically intertwined with brain connectivity and hence with the different network metrics taken under exam. Unfortunately, we do not have data about gyrification available, but we added a comment on how this issue could be an aspect to explore when studying brain network organization across brain volume. In doing so, we acknowledged not only works from Karl Zilles, but also from Pillay and Mota, as also suggested by Reviewer #3.

Discussion, page 13:

“*Finally, we observe that while the trends of connectome topology versus brain volume are statistically significant, there is still some residual variance among mammals with roughly equal brain size. This variance could be explained by differences between taxonomies, species, behavioral diversity, or ecological adaptations (Krubitzer, 1995). The different ways in which the cortex folds across species and orders might also explain why animals with the same brain volume present slightly different features of modular structure and network communication. According to the tension-based hypothesis (Van Essen, 1997, Van Essen 2020) indeed, gyrification is intrinsically intertwined with brain connectivity as the viscoelastic properties of the fiber tracts explain the folding of the brain in gyri and sulci. Furthermore, it has been demonstrated that the degree of cortical folding generally increases with brain size across mammals, but at a different scale between orders and families, and as a function of the cortical thickness in a way that thinner cortices appear more folded (Zilles, 2013; Pillay 2007; Mota 2015). Future studies where gyrification or cortical thickness data is available will be needed to uncover how much these factors impact variation of brain network organization across brain volume.*”

References:

Leah Krubitzer. “The organization of neocortex in mammals: are species differences really so different?” Trends in Neurosciences, 18(9):408–417, September 1995.

David C. Van Essen. “A tension-based theory of morphogenesis and compact wiring in the central nervous system.” Nature, 385(6614):313–318, January 1997. Number: 6614 Publisher: Nature Publishing Group.

Karl Zilles, Nicola Palomero-Gallagher, and Katrin Amunts. “Development of cortical folding during evolution and ontogeny.” Trends in Neurosciences, 36(5):275–284, May 2013.

Praneshri Pillay and Paul R. Manger. “Order specific quantitative patterns of cortical gyrification.” European Journal of Neuroscience, 25(9):2705–2712, 2007.

Bruno Mota and Suzana Herculano-Houzel. “Cortical folding scales universally with surface area and thickness, not number of neurons.” Science, 349(6243):74–77, July 2015. Publisher: American Association for the Advancement of Science.

------------------------------------------------------------------------------------------------------------------------

Minor point: The citations in this paper do not follow a simple order (1, 2, 3, etc.), making it difficult to track the references within the manuscript.

We changed the order of the references so that now they are ordered by appearance.

------------------------------------------------------------------------------------------------------------------------------------------------------------------------------------------------------------------------------------------------

REVIEWER 2

This is an interesting manuscript that addresses an important topic in evolutionary neuroscience - how differences in brain volume impact connectome topology. The authors use the MaMI database to identify connectivity modules and examine how connectivity features relate to this modular structure and change with brain size. The authors report that as brain size increases, modules become more compact and exhibit more costly connections, and that distance between notes progressively impacts their communication efficiency. I recommend major revisions to the manuscript, primarily due to the lack of consideration of phylogenetic relationships between species in their statistical analyses - I believe this change can be implemented by the authors. My comments are organized by section below:

We thank the Reviewer for their overall positive evaluation of the manuscript and for the suggestions they provided, to which we will respond hereafter.

------------------------------------------------------------------------------------------------------------------------

Introduction

* Clear and well-written

Results

* Clear and well-written - great descriptions of approach

We thank the reviewer for considering the manuscript clear and well-written.

------------------------------------------------------------------------------------------------------------------------

* Readers would benefit from added definitions of relevant terms in the first section (e.g., NSI, communicability, etc.), even though these are provided later in the section - definitions could also/alternatively be included in Figure 1

To make the paper more readable from the beginning, we added a glossary for the basic concept of network neuroscience and the network measures under exam. We also better described the same measure in Figure 1, panel A, as also suggested in a following comment.

Results, page 2:

*“Table 1. Glossary. Basic concepts and terms used in network neuroscience. More technical details about the network topological measures investigated are provided in the Methods section.”*

| *Networks and brain networks* | *A network is a mathematical object used to model complex systems, often biological, like the brain. Under the network conceptualization, the fundamental units of a system (cortical areas in the case of the brain) and their mutual interactions are formalized as a set of nodes connected (or not) by edges.* |
| --- | --- |
| *Network topology* | *Relation between nodes and edges. Network topology can be investigated through measures developed in the field of graph theory, like modularity and communication efficiency.* |
| *Network modules* | *Empirical networks, such as brain networks, often exhibit a non-random distribution of edges that results in distinct groups of highly interconnected nodes, also named modules, communities, or clusters. Network modules can be defined mathematically by optimizing modularity, a function that measures the strength of the division of the network into modules by comparing the within-module connectivity with a null model.* |
| *Network Communication* | *How nodes are integrated through sequences of edges, e.g., paths. Models of network communication range from routing protocols to diffusive processes. Cardinal measures falling in this range are: (i) shortest path efficiency, which captures the length of optimally efficient routes; (ii) search information, or the amount of information required from a random walker to travel along the shortest path, which estimates the accessibility of shortest routes in the network; (iii) communicability, or the sum of the lengths of all the possible paths connecting two nodes, which quantifies diffusive broadcasting.* |

------------------------------------------------------------------------------------------------------------------------

* In some areas, it would be helpful to provide a plain language interpretation of results (e.g., as brain size increases, the proportion of white matter increases faster than the proportion of grey matter, so larger brains are comprised of more white:grey matter; describe grey vs. white matter; etc.) -this is generally well-done throughout

We thank the reviewer for appreciating how we generally tried to use straightforward language to link variations in brain volume to variations in network topology. We further adopted this plain language in the first section of the Results, as suggested:

Results, page 3:

“*We obtained the following rule:* ${log}_{10} WM=\left( 1.16\pm0.0097 \right){log}_{10} GM+(-1.45\pm0.0445)$*, which means that as the brain size increases, the proportion of white matter grows faster than the proportion of grey matter.*”

------------------------------------------------------------------------------------------------------------------------

Figures

* Figure 1 - In its current form, this figure isn't super useful for comprehending what the different measures are capturing - I suggest the authors recreate this figure to add additional information

We thank the reviewer for this suggestion. We enriched Figure 1 so that now it includes schematics representation of the different measures and we hope this helps to better comprehend what they capture.

FIG. 1. *Schematic representation of the topological analysis*. In panel A the network topological measures under exams are displayed. On the left, modules of densely interconnected nodes are highlighted through different colors. On the right, three putative models to measure network communication efficiency between node *i* and node *j* are illustrated: shortest path efficiency (SPE), or communication via the shortest path; search information (NSI), which also quantifies the dispersion of the signal along the shortest path; communicability (CMY), or diffusive broadcast, where all the paths contribute to the network communication efficiency. In panels B and C are reported examples of adjacency and topological matrices for two mammals, the bat *Artibeus Jamacien* and the antelope *Orix*. On the left, we show the structural connectivity matrix (SC). Matrices used for the analysis of the modular structure are the co-classification matrix (CC), the adjacency matrix (SC), the cost matrix, and the matrix of Euclidean distances (ED). In this way, the probability of two nodes to being assigned to the same module can be compared with the weight and cost of the connection linking the two nodes and their physical distance across brain volume. The rows of these matrices have been reordered based on the consensus partition, whose modular borders are displayed as white lines. Matrices used for the analysis of communication efficiency are the Euclidean distance (ED), the shortest path efficiency (SPE), the negative search information (NSI), and the communicability (CMY) matrices. Thus, the physical distance between two nodes can be compared to the efficiency with which the signal is transmitted between them according to the three different models and across brain volume. Since all matrices are symmetrical, only their upper triangle is shown.

------------------------------------------------------------------------------------------------------------------------

* Figure 2 - Please color code the dots in A and B as in C; It would be helpful to add a plot showing white matter volume ~ brain volume

As suggested, we added another panel where we display the trend of white matter volume against the brain volume. However, because many points overlap in panels A and B, if we colored all the points many colors would be obscured. Thus, we changed those panels by adding colored squares in correspondence with the mean values of the distributions of the orders. The colors used are the same as in the panel reporting the brain volumes grouped by orders (now panel D) and in the following figures.

FIG 2. *Scaling laws of the relative sizes of grey matter and white matter volumes in the mammalian brain*. In panels A, B, and C the relative trends of grey matter, white matter, and total brain volumes are reported; for all three volumes, their logarithmic values have been displayed. The colored squares indicate the mean value for each order (colors are matched with panel D). In panel D the distribution of brain volumes within each taxonomic order is shown. Colored dots refer to single mammals, whereas white dots correspond to the mean value of the distributions. For clades comprising only one mammal the mean value was not reported. Clades are ordered on the x-axis from lowest to highest average brain volume: [2.64; 3.11; 3.25; 3.48; 3.88; 4.02; 4.15; 4.37; 4.59; 4.75; 5.18; 5.53]^10^mm^3^.

------------------------------------------------------------------------------------------------------------------------

Discussion

* Clear and well-written

* How do these results relate to established hypotheses of brain evo-devo (e.g., the tethering hypothesis: Buckner and Krienen 2013, Trends in Cog Sci)?

With the tethering hypothesis, Buckner and Krienen speculate on how the organization of the mammalian cortex changed when expanding from the primate mammalian ancestor to non-human primates and humans. These studies on the evolution of the primate brain are usually carried out to answer the questions: how did the mammalian brain evolve to accommodate areas underpinning human capabilities? How does a larger brain enable complex cognitive functions? The tethering hypothesis looks for an answer and focuses on how the scaling of the brain volume carried a shift in the predominant circuits, from primarily being linked to sensory-motor hierarchies to interconnected association networks that span the cortex.

Unfortunately, our work is not ideal for additional insight or speculation about this theory, for two reasons. First, the MaMI dataset does not include the human connectome. Second, a limitation of the dataset concerns the definition of the nodes. Because we did not have an anatomical template for each species in the dataset, and we wanted to carry out a comparative analysis on networks with the same number of nodes, we opted for a parcellation scheme based on k-means clustering of tractography streamlines. This procedure limits the identification of nodes in the cortex in that they cannot be directly compared or matched across species, and we do not know which areas they subtend (sensory, motor, visual, association, etc.). For these reasons, we cannot establish a relationship between increasing brain volume and the distribution of sensorimotor versus association areas in the cortex. However, in the revised version of the manuscript, we commented on how overcoming this limitation might help inform evolutionary theories like the one highlighted by the reviewer.

Discussion, page 12:

“*The main limitation of the study concerns the definition of nodes and edges in the reconstructed brain networks. An ideal comparative analysis would benefit from node correspondence across species, in order to localize and track changes in connectome topology. However, establishing homologies in brain regions of different mammals whilst accommodating evolutionary specialization is a work in progress (Krubitzer, Annals of the New York Academy of Science, 2009; Mars et al., eLife, 2018; Warrington et al., Science Advances, 2022). Moreover, while most of the efforts in this direction focus on human, macaque, and rat brains, there are many other species in the MaMI dataset which lack canonical or widely accessible anatomical templates. Here, we partially addressed this issue by applying the same parcellation methodology to each mammal. However, while we obtained brain networks with the same number of nodes, brain parcels have been identified through a stochastic procedure based on k-means that impacts node location on the cortex and hence edge weights. Thus, we could only examine global properties of modular organization and communication efficiency, without identifying specific neuroanatomical regions that drive changes in connectome topology associated with variations in brain volume. Moreover, because we cannot associate specific brain areas to nodes (i.e., sensory-motor, visual, etc.), we cannot speculate on how our results resonate with established evolutionary theories, like the tethering theory proposed in (Buckner and Krienen, Trends in Cognitive Science, 2013), where changes in brain organization have been associated with the scaling of brain volume in the primate brain. The challenge of node definition, together with other limitations related to white matter tracts reconstruction, and hence edge weight interpretation, have been extensively discussed in (Faskowitz et al., Frontiers in Neuroscience, 2023)*”

References:

Leah Krubitzer. “In Search of a Unifying Theory of Complex Brain Evolution.” Annals of the New York Academy of Sciences, 1156(1):44–67, 2009.

Rogier B Mars, Stamatios N Sotiropoulos, Richard E Passingham, Jerome Sallet, Lennart Verhagen, Alexandre A Khrapitchev, Nicola Sibson, and Saad Jbabdi. “Whole brain comparative anatomy using connectivity blueprints.” eLife, 7:e35237, May 2018.

Shaun Warrington, Elinor Thompson, Matteo Bastiani, Jessica Dubois, Luke Baxter, Rebeccah Slater, Saad Jbabdi, Rogier B. Mars, and Stamatios N. Sotiropoulos. “Concurrent mapping of brain ontogeny and phylogeny within a common space: Standardized tractography and applications.” Science Advances, 8(42):eabq2022, October 2022.

Randy L. Buckner and Fenna M. Krienen. “The evolution of distributed association networks in the human brain.” Trends in Cognitive Sciences, 17(12):648–665, December 2013. Publisher: Elsevier.

Joshua Faskowitz, Maria Grazia Puxeddu, Martijn P. van den Heuvel, Bratislav Misic, Yossi Yovel, Yaniv Assaf, Richard F. Betzel, and Olaf Sporns. “Connectome topology of mammalian brains and its relationship to taxonomy and phylogeny.” Frontiers in Neuroscience, 16, 2023.

------------------------------------------------------------------------------------------------------------------------

Methods

* Species that are more closely related to each other are more likely to be similar to each other - this observation is actually noted in the authors' descriptions of previous analyses of this dataset in the Introduction. Accordingly, phylogenetic comparative methods are necessary to control for the lack of independence (i.e., due to evolutionary relatedness) between data points (i.e., species). These methods can and should be implemented here, including:

-- Phylogenetic correlations (e.g., to replace the reported brain volume vs. grey matter volume correlation)

http://blog.phytools.org/2017/08/pearson-correlation-with-phylogenetic.html

-- Phylogenetic generalized least squares regression modeling (e.g., to replace regressions of grey matter volume ~ brain volume; modular organization measures ~ brain volume; etc.)

The authors can use e.g., the phytools R package and incorporate a mammal-wide phylogenetic tree (e.g., TimeTree), although some effort will likely be required to match species names between the dataset and tree; I would also consider running analyses with order included as a predictor to account for / identify potential inter-order variation in scaling relationships

Here the reviewer raises the question of what role phylogeny plays in our results. Given the richness and diversity of our dataset, this is a natural question to pose. Indeed, previous work, carried out on the same dataset, has extensively examined how phylogeny impacts connectome topology (Faskowitz et al., Frontiers in Neuroscience, 2023; Suarez et al., eLife, 2022). These previous results showed that connectome topology is more similar among species belonging to the same order. In the present study, we intended to focus on how brain volume – instead of phylogeny – impacts on connectome topology. However, we acknowledge that there are some potential interactions between brain volume and phylogeny. As shown in Figure 2D, brain volume and orders are collinear: on average different orders display different brain volumes, although the inter-order variability is high above all among Chiroptera, Rodentia, Carnivora, Primates, and Artiodactyla, and brain volume overlaps across these orders. Following the reviewer’s suggestion, to confirm the robustness of our results and demonstrate that connectome topology is shaped by brain volume, we carried out a supplementary analysis which we comment on in the Discussion section. In this further analysis, we computed the partial correlations between modularity/communication indices and brain volume controlling for (i) the similarity across species and (ii) orders.

The similarity across species, or phylogenetic distances, have been estimated stochastically for the MaMI database (Faskowitz et al., 2023) using a set of 10,000 phylogenetic trees inferred using a Bayesian approach (Upham et al., 2019). For each tree, we computed the pairwise distance between species by adding the patristic distance along the tree branches. Then, we pruned distance matrices to match the species contained in the MaMI database (for more details see Faskowitz et al., 2023). As a result, we have 10,000 matrices containing phylogenetic distances among the species. By taking the centroid of these matrices and computing a Principal Component Analysis (PCA) we obtained coefficients that encode modes of covariance between species. We used the first three coefficients (explaining most of the variance in the PCA) as predictors in the partial correlation between modularity/communication indices and brain volume. The results are reported in Supplementary Information, S6.

The results that we obtained show that, while in some cases they are attenuated, the correlations documented in the main analysis hold. This indicates that phylogenetic distances and taxonomy orders only partially explain the observed trends. Again, the fact that these trends are partially explained by phylogeny is not surprising given the collinearity observed in Figure 2D between brain volume and taxonomy.

Discussion, page 13:

“*As shown in Figure 2D, brain volume and orders are collinear: on average different orders display different brain volumes, although the inter-order variability is high above all among Chiroptera, Rodentia, Carnivora, Primates, and Artiodactyla, and brain volume overlaps across these orders. For this reason, we might expect that the trends here commented could be partially explained by taxonomies and phylogeny. To confirm the robustness of our results and prove that connectome topology is shaped by brain volume, we carried out a supplementary analysis where we computed the partial correlations between modularity/communication indices and brain volume controlling first for the similarity across species and then for taxonomy orders. While several packages are available to estimate phylogeny trees and distances (e.g., Revell, Methods in Ecology and Evolution, 2012; Revell, bioRxiv, 2023), we build this analysis on our earlier work (Faskowitz et al., Frontiers in Neuroscience, 2023), where phylogenetic distances were estimated for the species contained in the MaMI database. We report these control analyses in Supplementary Information, S6. Our results show that, while in some cases they are attenuated, the correlations between network metrics and brain volume remain significant even after phylogenetic distances are accounted for. This indicates that phylogeny and taxonomy only partially explain the observed relationships. Again, this outcome is not surprising given the collinearity observed in Figure 2D between brain volume and taxonomy.*”

Supplementary Information, S6:

“*S6. Controlling correlations with similarity between species and orders.*

*As shown in Figure 2D, brain volume and orders are collinear: on average different orders display different brain volumes, although the inter-order variability remains high above all among Chiroptera, Rodentia, Carnivora, Primates, and Artiodactyla, and brain volume overlaps across these orders. To confirm the robustness of our results and prove that connectome topology is shaped by brain volume, we carried out this further analysis where we computed the partial correlations between modularity/communication indices and brain volume controlling first for the similarity across species and then for orders. The results are shown in Figures S5 and S6, respectively.*

*The similarity across species, or phylogenetic distances, have been estimated stochastically for the MaMI database (Faskowitz et al., 2023) using a set of 10,000 phylogenetic trees inferred using a Bayesian approach (Upham et al., 2019). For each tree, we computed the pairwise distance between species by adding the patristic distance along the tree branches. Then, we pruned distance matrices to match the species contained in the MaMI database. For more details see (Faskowitz et al., 2023). As a result, we have 10,000 matrices containing phylogenetic distances among the species. By taking the centroid of these matrices and computing a Principal Component Analysis (PCA), we obtained coefficients that encode modes of covariance between species. We used the first three coefficients (explaining most of the variance in the PCA, see Figure S5, panel A) as predictors in the partial correlation between modularity/communication indices and brain volume. As a result, we observed that the correlations documented in the main text hold.* *In fact, p-values and correlation coefficients (Figure S5, panel B) remain consistent with those reports in Figures 3 and 4.*

*We repeated an analogous partial correlation analysis but this time controlling for taxonomy orders. Results are reported in the tables in Figure S6. All the correlations remain statistically significant (p-value<0.05), although the correlation coefficients are weaker. The only exception is given by the partial correlation between intra-module density and brain volume, which is non-significant.*

*Altogether, these results show that, while in some cases they are attenuated, the correlations documented in the main analysis hold. This demonstrates that phylogenetic distances and orders only partially explain the trends. Again, the fact that these trends are partially explained by phylogeny is not surprising given the collinearity observed in Figure 2D between brain volume and taxonomy.*

*Figure S5. Results of the partial correlations between modularity/communication indices and brain volume using as predictors the similarity among species. In panel A we report the explained variance of the PCA, whereas in panel B the correlation coefficients and p-values.*

*Figure S5. Results of the partial correlations between modularity/communication indices and brain volume using as predictors the taxonomy orders.*”

References:

Joshua Faskowitz, Maria Grazia Puxeddu, Martijn P. van den Heuvel, Bratislav Misic, Yossi Yovel, Yaniv Assaf, Richard F. Betzel, and Olaf Sporns. “Connectome topology of mammalian brains and its relationship to taxonomy and phylogeny.” Frontiers in Neuroscience, 16, 2023.

Laura E Suarez, Yossi Yovel, Martijn P van den Heuvel, Olaf Sporns, Yaniv Assaf, Guillaume Lajoie, and Bratislav Misic. “A connectomics-based taxonomy of mammals.” eLife, 11:e78635, November 2022. Publisher: eLife Sciences Publications, Ltd.

Upham, N. S., Esselstyn, J. A., and Jetz, W. (2019). „Inferring the mammal tree: Species-level sets of phylogenies for questions in ecology, evolution, and conservation.” PLoS Biol. 17:e3000494. doi: 10.1371/journal.pbio.3000494.

Liam J. Revell. “phytools: an R package for phylogenetic comparative biology (and other things).” Methods in Ecology and Evolution, 3(2):217–223, 2012. eprint: <https://onlinelibrary.wiley.com/doi/pdf/10.1111/j.2041-210X.2011.00169.x>.

Liam J. Revell. “phytools 2.0: An updated R ecosystem for phylogenetic comparative methods (and other things)”, August 2023. Pages: 2023.03.08.531791 Section: New Results.

------------------------------------------------------------------------------------------------------------------------

I hope the authors find my comments useful in revising their manuscript.

We thank the reviewer because we did find their comments useful and we believe our manuscript improved in clarity and robustness.

------------------------------------------------------------------------------------------------------------------------------------------------------------------------------------------------------------------------------------------------

REVIEWER 3

~~~~~~~~~~Summary~~~~~~~~~~

In general, comparative analysis of brain networks across species is greatly limited by the difficulty in alignment of nodes across species, that is, the designation of homologous regions. In their innovative study, the authors have bypassed this issue by focusing on network measures which are agnostic to the identities of individual nodes. Broadly, the claim is made that structural brain networks become more modular with increasing size. This hypothesis is logical giving the increasing volumetric constraints and metabolic burdens of non-modular wiring with size, but to date has not been adequately tested.

To examine this question, the authors performed diffusion MRI fiber tractography (previously reported) on ~200 ex-vivo examples of ~100 species from 12 orders. Mesoscale nodes were derived by clustering these tracts and the relationships, specifically module cohabitation probability, among nodes quantified by subsequent multi-scale community detection. The amount of variance in module cohabitation of two nodes explained by the volumetric and metabolic cost of the connection between them appears strongly predictive total brain volume. Similarly, brain volume appears strongly predictive of the amount of variance in internodal communicative capacity explained by internodal distance and the amount of variance in internodal module cohabitation explained by internodal communicability.

In whole the results shown support the author's claims. The analyses performed are rigorous, figures mostly clear, and the abstract measures well explained. My first two comments concern principled quantitative adjustments that I do not anticipate will dramatically change the tenor of the results. The third presents an alternative to the volumetric hypothesis which may call for additional analyses and certainly calls for additional discussion. The remaining comments highlight underexamined elements for further discussion.

~~~~~~~~~~Major concerns~~~~~~~~~~

1. It is unusual to calculate correlation coefficients of correlation coefficients. One reason is that the sample distributions of coefficients become progressively skewed as the mean coefficient approaches 1 or -1. This can be resolved by applying Fisher's z-transformation to primary coefficients before correlating them with brain volume to yield secondary coefficients. While this adjustment is principled and should be performed, I do not believe it will change the study's findings, as the reported primary coefficients are clustered reasonably tightly and do not approach the [-1 1] limits closely, and because the secondary correlations were calculated with the robust Spearman rank-order procedure.

We thank the reviewer for this valuable methodological suggestion. In the reviewed version of the manuscript, we z-transformed the distributions of the correlation coefficients and re-computed the correlations with brain volume. As expected, while the extreme points slightly change location in the xy-plane, all the correlations hold and are confirmed. Here we report the new figures:

FIG. 3. **Modular organization changes with brain volume**. In the six panels, we represented the distributions of brain volume (x-axis) versus six indices that characterize the modular structure: (A) correlation between co-classification probability and weight of the connection *ρ*(CC,weight); (B) correlation between co-classification probability and Euclidean distance *ρ*(CC,ED); (C) correlation between co-classification probability and cost of the connection *ρ*(CC,cost); (D) within-module density, i.e., average number of connections linking nodes participating in the same modules normalized by module size; (E) fraction of long-distance connections used to link nodes belonging to different modules; (F) co-classification probability of nodes belonging to different hemispheres. In each panel, the different taxonomies are highlighted through different colors, and the median values of their respective measures are reported with squares of the same color.

FIG. 4. **Network communication efficiency changes with brain volume.** In panels A-C, we represented the distributions of brain volume (x-axis) versus four indices that characterize communication efficiency in the brain networks: (A) correlation between Euclidean distance and shortest path efficiency, *ρ*(ED,SPE); (B) correlation between Euclidean distance and negative search information, *ρ*(ED,NSI); (C) correlation between Euclidean distance and communicability, *ρ*(ED,CMY.). In panels D and E we report the mutual trend of SPE_scaled_ and CMY_scaled_ once scaled in a common space and their trend with respect to brain volume. In each panel, the different taxonomies are highlighted through different colors, and the median values of their respective measures are reported with squares of the same color.

FIG. 5. **Bridges between the modular organization and communication efficiency**. In the three panels, we represented the distributions of brain volume (x-axis) versus the correlation between co-classification probability and the three indices that characterize the communication efficiency in the brain networks: SPE, NSI, and CMY. In each panel, the different taxonomies are highlighted through different colors, and the median values of their respective measures are reported with squares of the same color.

------------------------------------------------------------------------------------------------------------------------

2. It is unclear why the Euclidean distance between nodes was used when inter-node fiber length was estimated and used for the edge cost calculation. Fiber length is surely a more biologically meaningful proxy for the closeness of node and modules, especially as, at least in macaques, fiber length is not anticorrelated to conduction velocity (Innocenti et al., 2014). Furthermore, in gyrencephalic cortices, Euclidean paths often implausibly transect sulci. Summarily, when examining communication measures (Fig. 4), cost rather than Euclidean distance alone seems more appropriate, though given the similarity of Figs. 3 A, B, C the conclusions will almost certainly hold.

Here the reviewer introduces a critical observation. There are two main reasons why we used Euclidean distance instead of fiber length. First, anatomical brain networks reconstructed with diffusion imaging and tractography are not fully connected, so fiber length is not available for every pair of nodes. The second reason is more conceptual. While we could have restricted the analysis only to anatomically connected nodes for which we had an estimate of the fiber length, this would have limited the scope of our network communication analyses. Network communication measures have been introduced to quantify how network topology underpins inter-areal integration of information among both anatomically connected and non-connected brain regions (Avena-Konisberger et al., 2018). In fact, while anatomically connected nodes can communicate via direct connections, signaling between unconnected nodes relies on polysynaptic communication which can be modeled through different paradigms of network communication ranging from routing to diffusion. These models have been reported to be especially powerful in explaining functional connectivity among anatomically disconnected nodes, validating the hypothesis that inter-areal integration of information is mediated by the network topology (Goni et al., 2014; Zamani-Esfahlani et al., 2022; Seguin et al., 2023).

For these reasons, it makes more sense to model network communication between every pair of nodes, not just between those anatomically connected for which we have a measure of fiber length and observe how communication efficiency changes with nodes physical distance, and ultimately how this relationship is shaped by brain volume across mammals.

We added these considerations in the Results section, page 8:

“*Note that in this analysis we used ED as a measure of distance between nodes, instead of tract length, for two reasons. First, anatomical brain networks reconstructed with diffusion imaging and tractography are not fully connected, so fiber length is not available for every pair of nodes. The second reason is more conceptual. While we could have restricted the analysis only to anatomically connected nodes for which we had an estimate of the fiber length, this would have limited the scope of our network communication analyses. Network communication paradigms have been introduced to model and quantify how network topology underpins inter-areal integration of information among both anatomically connected and non-connected brain regions, whose signaling relies on polysynaptic paths [24].*”

References:

Andrea Avena-Koenigsberger, Bratislav Misic, and Olaf Sporns. “Communication dynamics in complex brain networks.” Nature Reviews Neuroscience, 19(1):17–33, January 2018.

Joaquın Goni, Martijn P. van den Heuvel, Andrea Avena-Koenigsberger, Nieves Velez de Mendizabal, Richard F. Betzel, Alessandra Griffa, Patric Hagmann, Bernat Corominas-Murtra, Jean-Philippe Thiran, and Olaf Sporns. “Resting-brain functional connectivity predicted by analytic measures of network communication.” Proceedings of the National Academy of Sciences, 111(2):833–838, January 2014.

Farnaz Zamani Esfahlani, Joshua Faskowitz, Jonah Slack, Bratislav Misic, and Richard F. Betzel. “Local structure-function relationships in human brain networks across the lifespan.” Nature Communications, 13(1):2053, April 2022.

Caio Seguin, Maciej Jedynak, Olivier David, Sina Mansour, Olaf Sporns, and Andrew Zalesky. “Communication dynamics in the human connectome shape the cortex wide propagation of direct electrical stimulation.” Neuron, March 2023.

------------------------------------------------------------------------------------------------------------------------

3. There is no discussion of the differing degrees of gyrification among the examined taxa. Given that that gyrification is correlated to brain weight (and presumably volume) across species (Mota & Herculano-Houzel, 2015; Pillay & Manger, 2007), is it possible the observed trends are driven by or at least modulated by gyrification. Would the figures be similar if an index of gyrification was substituted for volume? There is considerable variation in the network metrics at a given brain volume; can some of it be explained by gyrification?

We thank the reviewer for suggesting that we comment on our results in light of gyrification, a point also made by Reviewer #1. Gyrification indeed is intrinsically intertwined with brain connectivity and hence with the different network metrics taken under exam. In this revised version of the manuscript, we commented on how this could be a factor to take into consideration when studying brain network organization across brain volume. In doing so, we acknowledged works from Karl Zilles, Pillay, and Mota, as also suggested by Reviewer #1.

Discussion, page 13:

“*Finally, we observe that while the trends of connectome topology versus brain volume are statistically significant, there is still some residual variance among mammals with roughly equal brain size. This variance could be explained by differences between taxonomies, species, behavioral diversity, or ecological adaptations (Krubitzer, 1995). The different ways in which the cortex folds across species and orders might also explain why animals with the same brain volume present slightly different features of modular structure and network communication. According to the tension-based hypothesis (Van Essen, 1997) indeed, gyrification is intrinsically intertwined with brain connectivity as the viscoelastic properties of the fiber tracts explain the folding of the brain in gyri and sulci. Furthermore, it has been demonstrated that the degree of cortical folding generally increases with brain size across mammals, but at a different scale between orders and families, and as a function of the cortical thickness in a way that thinner cortices appear more folded (Zilles, 2013; Pillay 2007; Mota 2015). Future studies where gyrification or cortical thickness data is available will be needed to uncover how much these factors impact variation of brain network organization across brain volume.*”

References:

Leah Krubitzer. “The organization of neocortex in mammals: are species differences really so different?” Trends in Neurosciences, 18(9):408–417, September 1995.

David C. Van Essen. “A tension-based theory of morphogenesis and compact wiring in the central nervous system.” Nature, 385(6614):313–318, January 1997. Number: 6614 Publisher: Nature Publishing Group.

Karl Zilles, Nicola Palomero-Gallagher, and Katrin Amunts. “Development of cortical folding during evolution and ontogeny.” Trends in Neurosciences, 36(5):275–284, May 2013.

Praneshri Pillay and Paul R. Manger. “Order specific quantitative patterns of cortical gyrification.” European Journal of Neuroscience, 25(9):2705–2712, 2007.

Bruno Mota and Suzana Herculano-Houzel. “Cortical folding scales universally with surface area and thickness, not number of neurons.” Science, 349(6243):74–77, July 2015. Publisher: American Association for the Advancement of Science.

------------------------------------------------------------------------------------------------------------------------

4. The control analyses performed for random networks and fixed network density are welcome. Is there any relationship between un-thresholded network density and brain volume, and how do the unthresholded densities quantitatively compare to the thresholds? Given that long distance connections are much weaker than short range connections, one would expect thresholding to increase modularity. The fact that key findings are not affected by imposing very strict density threshold requires further explanation.

We thank the reviewer for acknowledging our efforts in making control analyses considering different null models and network densities. To answer their question, we observed no correlation between brain volume and network density in the un-thresholded networks, as reported in the figure below.

We argue that it is the relation between modularity features and brain volume that holds across different levels of thresholding. This does not mean that the specific modularity indices do not change. However, they change in the same way across mammals, so that their correlation with brain volume persists. This makes our key findings robust to different connection density thresholds. Moreover, while it is true that on average longer connections are weaker and thus prone to be discarded when decreasing the density, an important aspect of the thresholding that we performed is that it has been made retaining a backbone of edges forming a connected component. This procedure ensures that thresholding does not break the network into disconnected components and usually preserves a percentage of long-distance connections.

We added this consideration in the main text, in the Results section, page 7:

“*Another validation consisted of computing the correlations also on thresholded networks and with respect to grey and white matter volumes, instead of total brain volume. Results are consistent with those shown above and have been reported in Supplementary Information, Table 1. This does not mean that modularity indices do not change in networks with different densities, but presumably, they change consistently across mammals, so that their relation with brain volume holds.*”

------------------------------------------------------------------------------------------------------------------------

5. Could the authors speculate on why some the species appear to markedly deviate from the observed trends? For example, there appears to be one marsupial and one chiropteran which stand out in their modularity and communication efficiency. They may have unique anatomical or ecological adaptations which may serve as enriching counterexamples.

This is an interesting observation. The fact that some species appear to deviate from the observed trends is indeed interesting but also expected. In fact, we do not hypothesize that brain volume fully explains connectome topology. Rather, as the reviewer suggested, we might find differences between species with equal brain size that are due to anatomical, behavioral, or ecological adaptations. We briefly discussed this aspect in the original manuscript and now we expanded these considerations with respect to gyrification. We added a paragraph where we comment on why some species deviate from the reported trends. However, we want to emphasize our confidence that our results are robust. In fact, the observed deviations do not affect the significance of the correlations, which means that brain volume largely explains the differences in the network topology that we commented on in the manuscript.

We refer to this observation in the Discussion section, page 13, from “*Finally, we observe …*” to “*across brain volume*” (it is the same paragraph reported as an answer to comment #3).

------------------------------------------------------------------------------------------------------------------------

6. The insensitivity of MRI tractography to the directionally of connections is well known. Does the possible asymmetry of edges have implications when discussing routing- vs diffusion- signaling strategies? Prima facie, it seems obscuring true edge asymmetry would bias toward diffusive strategies.

The reviewer is right that through MRI tractography we can only reconstruct undirected anatomical brain networks, and that network communication measures might change when considering directed networks instead. However, we cannot think of any mathematical reason supporting the idea that edge asymmetry would hinder routing more than diffusive processes. Given two nodes *i* and *j*, the paths connecting them might result longer no matter if they are used to compute the shortest path, communicability, or other communication measures based on routing or diffusion.

Furthermore, we speculate that our results are robust in relation to edge directionality. In fact, previous studies showed that there is a high correlation between the asymmetry computed on communication matrices obtained from directed and undirected brain networks. In (Seguin et al., Nature Communication, 2019) the authors considered the directed connectomes of drosophila, mouse, and macaque reconstructed through light-microscopy and tract tracing, and computed network communication matrices. After symmetrizing the three networks, they compute network communication matrices on both directed and undirected connectomes. All the communication matrices were asymmetric. The authors found a significant correlation between the asymmetry observed in the communication matrices computed on directed and undirected connectomes. However, while in the former the asymmetry is induced by both edge directionality and communication measures, in the latter it only comes from communication measures. This result suggests that even when undirected, the topology of the connectome is influential in determining the directionality of neural signaling in the absence of directed connections.

We comment on this in the discussion section, page 12:

“*We speculate that, while we built our network communication analyses on undirected networks, the results that we presented are robust in relation to edge directionality, for two reasons. First, there are not mathematical reasons for which one communication measures should be hampered more than the others in directed networks. Second and most important, previous studies demonstrated that even when undirected, the topology of the connectome is pivotal in determining the directionality of information transmission in absence of directed connections [65].*”

References:

Caio Seguin, Adeel Razi, and Andrew Zalesky. “Inferring neural signaling directionality from undirected structural connectomes.” Nature Communications, 10(1):4289, September 2019.

------------------------------------------------------------------------------------------------------------------------

7. While the relationship between mesoscale structural and functional connectivity is fraught (Honey et al., 2009), what functional predictions can be made from result from authors' findings, e.g., "wiring facilitates signal broadcasting in bigger brains and selective transmission of information in smaller brains". Is there evidence for these predictions in the mouse and human functional literature (being better sampled examples of small and big brains). Similarly, how does anatomical spatial compactness square with wide spatially noncontiguous functional networks, e.g., default mode and language processing (Ji et al., 2019)?

Unfortunately, we do not have data from which we can estimate functional connectivity in the MaMI dataset, so the answer we can give to this comment is purely speculative. We know that there is growing evidence that network communication models can explain inter-areal functional connectivity (Goni et al., PNAS, 2014; Seguin et al., Network Neuroscience, 2020; Zamani-Esfahlani, Nature Communication, 2022). Hence, while it is true – as the Reviewer states – that the relationship between anatomical and functional connectivity is fraught, previous literature suggests that network communication is useful to bridge the gap between the two. However, the comparison of routing versus diffusion processes is fairly recent in network neuroscience, as most of the studies on human and non-human mammals have been focusing primarily on measures of shortest paths (i.e., routing measures). Only recently, a few studies started to address this issue. In (Greyson et al., Neuron, 2016) the authors demonstrate that diffusion-based network communication (recapitulated by communicability) is suitable for approximating functional connectivity in the primate brain. In (Seguin et al., Neuron, 2023) the authors directly compare routing and diffusion models on human brain networks and show that the latter better explains signal propagation after stimulation through white matter. These two studies present evidence that diffusion models are well-suited for large brains. In (Griffa et al., bioRxiv, 2022) the authors analyzed rat, macaque, and human brain data and they indicate that increasing species complexity and brain volume is associated with a shift of communication policy, from routing to diffusion. Although we do not have functional data to confirm these findings, based on our results we can speculate that the topology of the anatomical network changes with increasing brain volume in order to accommodate diffusion-like signaling.

We better commented on this aspect in the Discussion section, page 12:

“*We speculate that brain volume directly shapes the wiring diagram of the mammalian brain in order to facilitate neural information transmission via signaling broadcasting in bigger brains and via selective pathways in smaller brains. Even if we do not have functional connectivity data to confirm what we speculate, this finding and its interpretation align with [68]. There, the authors analyzed rat, macaque, and human brain data and, by introducing a parallel communication score, they hypothesize that increasing species complexity and brain volume are associated with a shift of communication policy, from selective (i.e., routing-like) to parallel (i.e., diffusion-like). Moreover, in [69] and [67] it has been shown that diffusion-based models are well suited to explain functional connectivity and signal propagation after stimulation in non-human and human primates. The quest of routing versus diffusion is fairly new in network neuroscience. Our results contribute in demonstrating how the topology of anatomical brain networks changes with brain volume and how it underpins information transmission facilitating signaling diffusion and routing in large and small brains, respectively.*”

As for the second part of the Reviewer’s comment, we know that there is not a one-to-one relationship between edges of anatomical and functional connectivity, so we do not expect the modular structure of the two modalities to perfectly overlap (Puxeddu et al., NeuroImage, 2022). It could certainly be the case that not all compact anatomical modules result in compact functional modules. How can we make sense of this? Previous studies conducted by members of our team (Seguin et al., NeuroImage, 2022) showed that if we compute communication measures on structural networks and find modules in the resulting communication matrices, they better approximate functional modular structure. Again, this provides evidence that network communication is suitable to model how anatomical topology underpins polysynaptic information transmission and hence functional connectivity.

References:

Joaquın Goni, Martijn P. van den Heuvel, Andrea Avena-Koenigsberger, Nieves Velez de Mendizabal, Richard F. Betzel, Alessandra Griffa, Patric Hagmann, Bernat Corominas-Murtra, Jean-Philippe Thiran, and Olaf Sporns. “Resting-brain functional connectivity predicted by analytic measures of network communication.” Proceedings of the National Academy of Sciences, 111(2):833–838, January 2014.

Caio Seguin, Ye Tian, and Andrew Zalesky. “Network communication models improve the behavioral and functional predictive utility of the human structural connectome.” Network Neuroscience, 4(4):980 1006, November 2020.

Farnaz Zamani Esfahlani, Joshua Faskowitz, Jonah Slack, Bratislav Misic, and Richard F. Betzel. “Local structure-function relationships in human brain networks across the lifespan.” Nature Communications, 13(1):2053, April 2022.

David S. Grayson, Eliza Bliss-Moreau, Christopher J. Machado, Jeffrey Bennett, Kelly Shen, Kathleen A. Grant, Damien A. Fair, and David G. Amaral. “The Rhesus Monkey Connectome Predicts Disrupted Functional Networks Resulting from Pharmacogenetic Inactivation of the Amygdala.” Neuron, 91(2):453–466, July 2016.

Caio Seguin, Maciej Jedynak, Olivier David, Sina Mansour, Olaf Sporns, and Andrew Zalesky. “Communication dynamics in the human connectome shape the cortex wide propagation of direct electrical stimulation.” Neuron, March 2023.

Alessandra Griffa, Mathieu Mach, Julien Dedelley, Daniel Gutierrez-Barragan, Alessandro Gozzi, Gilles Allali, Joanes Grandjean, Dimitri Van De Ville, and Enrico Amico. “The evolution of information transmission in mammalian brain networks.” Technical report, bioRxiv, May 2022.

Maria Grazia Puxeddu, Joshua Faskowitz, Olaf Sporns, Laura Astolfi, and Richard F. Betzel. “Multi-modal and multi-subject modular organization of human brain networks.” NeuroImage, 264:119673, December 2022.

Caio Seguin, Sina Mansour L, Olaf Sporns, Andrew Zalesky, and Fernando Calamante. “Network communication models narrow the gap between the modular organization of structural and functional brain networks.” NeuroImage, 257:119323, August 2022.

------------------------------------------------------------------------------------------------------------------------

~~~~~~~~~~Minor concerns~~~~~~~~~~

The number of digits used for rho values throughout the manuscript is inconsistent.

All the correlation coefficients now have the same number of digits from Figure 3 to Figure 5. In Figure 2 \rho values have more digits to highlight the agreement with results previously reported (ref. 14, 40, 41 of the manuscript).

------------------------------------------------------------------------------------------------------------------------

Citation 82 needs to be updated from the preprint to the published article. It's probably a good idea to check the other preprint citations as well.

We thank the reviewer for checking the references. We updated reference 82 and also checked on all the other cited preprints.

------------------------------------------------------------------------------------------------------------------------

Fig. 1: The colormaps are diverging, but the mid-point of the colorscale is not meaningful (e.g., 0)

Indeed, the mid-points of the distributions In Figure 1 are not meaningful and diverging colormaps should not be used in this case, so we adopted sequential colormaps (blue to yellow for modularity measures and white to purple communication measures) taken from the GitHub package “BrewerMap” (https://github.com/DrosteEffect/BrewerMap).

------------------------------------------------------------------------------------------------------------------------

Fig. 3: Panels B and C should be reversed as weight and length are constituents of cost.

We reversed panels B and C in Figure 3.

------------------------------------------------------------------------------------------------------------------------

Fig. 4: The direction the SPE axis is reversed in panel D vs panel E. Also, unlike the CMY axis, the ticks are not consistent between the panels. Also, the descriptions of panels B and C are reversed in the legend relative to the to figure and the abbreviation "co." is used instead of "CMY".

We reversed the direction of SPE in panel E and changed its ticks so that now everything is consistent with the one in panel D. We also fixed the caption of the figure.

------------------------------------------------------------------------------------------------------------------------

Fig. 6: I know the cortices at the top of the figure are meant to be illustrative, but they overemphasize the effect of brain volume by (1) showing an under-scaled size difference, and (2) presenting the same shape and folding pattens as the same in the different scaled brains.

As the reviewer noted, figure 6 is meant to be illustrative, a schematic representation of the results of our work. For it to be readable we cannot respect the actual difference in brain volumes (the smallest brain would be extremely small and barely visible, and the biggest brain would exceed the size of the page). However, we added in the caption a sentence to specify that what we are reporting is a toy model, and in reality there are bigger differences in terms of brain volume and gyrification.

Page 11:

“*FIG. 6. Schematic representation of how modular organization changes across brain volume. In the upper half of the figure, we report a toy model showing how as the brain volume increases modules become more spatially compact and dense, comprising stronger and more costly connections. Long-distance connections are used to connect nodes from different modules rather than nodes co-participating in the same module. The number of inter-hemispheric modules does not change with brain volume. In the schematic that we propose the difference between brain volumes is under-scaled for readability purposes and the gyrification does not mean to reflect any particular species of the dataset. In the lower half of the figure, we display as an example the modular structure of two mammalian brains present in the dataset, one small (left) and the other large (right), belonging to the Chiroptera and Artiodactyla orders, respectively.*”

------------------------------------------------------------------------------------------------------------------------

There are a few very minor typos / grammatical errors in the manuscript, e.g., not pluralizing 'cost' in the second sentence of the abstract, substituting 'tour' for 'our' in the first paragraph of Pg.6 Col. 2, substituting 'constrains' for 'constraints' twice in the last paragraph of Pg. 11 Col. 1.

We proofread the manuscript and corrected the minor typos.

------------------------------------------------------------------------------------------------------------------------

~~~~~~~~~~References~~~~~~~~~~

Honey, C. J., Sporns, O., Cammoun, L., Gigandet, X., Thiran, J. P., Meuli, R., & Hagmann, P. (2009). Predicting human resting-state functional connectivity from structural connectivity. Proceedings of the National Academy of Sciences of the United States of America, 106(6), 2035-2040. https://doi.org/10.1073/pnas.0811168106

Innocenti, G. M., Vercelli, A., & Caminiti, R. (2014). The Diameter of Cortical Axons Depends Both on the Area of Origin and Target. Cerebral Cortex, 24(8), 2178-2188. https://doi.org/10.1093/cercor/bht070

Ji, J. L., Spronk, M., Kulkarni, K., Repovš, G., Anticevic, A., & Cole, M. W. (2019). Mapping the human brain's cortical-subcortical functional network organization. NeuroImage, 185(September 2018), 35-57. https://doi.org/10.1016/j.neuroimage.2018.10.006

Mota, B., & Herculano-Houzel, S. (2015). Cortical folding scales universally with surface area and thickness, not number of neurons. Science, 349(6243), 74-77. https://doi.org/https://doi.org/10.1126/science.aaa9101

Pillay, P., & Manger, P. R. (2007). Order‐specific quantitative patterns of cortical gyrification. European Journal of Neuroscience, 25(9), 2705-2712. https://doi.org/https://doi.org/10.1111/j.1460-9568.2007.05524.x
